# Potential Role of Amino Acid/Protein Nutrition and Exercise in Serum Albumin Redox State

**DOI:** 10.3390/nu10010017

**Published:** 2017-12-24

**Authors:** Yasuaki Wada, Yasuhiro Takeda, Masashi Kuwahata

**Affiliations:** 1 Wellness & Nutrition Science Institute, Morinaga Milk Industry Co., Ltd., 51-83 Higashihara, Zama, Kanagawa-Pref. 252–8583, Japan; ya_taked@morinagamilk.co.jp; 2 Departments of Nutrition Science, Graduate School of Life and Environmental Sciences, Kyoto Prefectural University, 1-5 Shimogamo-hangi-cho, Sakyo, Kyoto 606–8522, Japan; kuwahata@kpu.ac.jp

**Keywords:** amino acids, exercise, protein, oxidative stress, redox state of serum albumin, skeletal muscle

## Abstract

Albumin is the major protein in the serum of mammals. It is synthesized exclusively in the liver, before being secreted into the circulation. Similar to skeletal muscle protein, albumin synthesis is stimulated by dietary amino acids and proteins as well as exercise. Albumin has three isoforms based on the redox states of the free cysteine residue at position 34. The redox state of serum albumin has long been extensively investigated in terms of oxidative stress-related chronic diseases, with the redox state of serum albumin having been regarded as a marker of systemic oxidative stress. However, according to recent animal studies, the redox state of serum albumin is modulated by albumin turnover and may also reflect amino acid/protein nutritional status. Furthermore, as the redox state of serum albumin is modulated by exercise training, measuring the pre- and post-exercise redox states of serum albumin in athletes may be useful in assessing amino acid/protein nutritional status and exercise-induced oxidative stress, which are closely associated with skeletal muscle adaptive responses. This article extensively reviews serum albumin and the redox state of albumin in the context of amino acid/protein nutritional status and exercise training.

## 1. Introduction

Skeletal muscle tissue has an enormous potential in responding to exercise, such as being involved in stimulation of muscle protein synthesis and mitochondrial biogenesis [1,2]. However, exercise also stimulates muscle protein breakdown, and the net protein balance remains negative in the absence of nutrient intake [1]. Exercise is also accompanied by other consequences, such as glycogen depletion [3] and possibly muscle damage, which manifests as muscle soreness and impaired muscle function [4,5,6]. The intake of nutrients, especially amino acids and protein, are indispensable for potentiating positive effects induced by exercise. Dietary amino acids and proteins elevate muscle protein synthesis and suppress muscle protein breakdown, leading to positive net protein balance [7,8,9]. These nutrients may augment muscle mass accretion primarily via improvement in intracellular amino acid availability [10]. Branched-chain amino acids (BCAA) and/or leucine (LEU) have a particular role in the facilitation of muscle protein synthesis through mammalian target rapamycin (mTOR) signaling pathway [11,12]. It has been shown in animal models that dietary BCAA augments exercise-induced mitochondrial biogenesis [13], which likely contributes to an improvement in energy metabolism. Protein ingestion potentiates post-exercise glycogen recovery particularly when co-ingested with sub-optimal amounts of carbohydrates [14]. The insulinogenic effect of protein may be responsible for the recovery of muscle tissue. In the context of recovery from exercise-induced muscle damage, Leu-enriched essential amino acids attenuated muscle soreness and enhanced muscle repair in rats [15,16], while a recent systematic review concluded that BCAA may only be effective in alleviating low-to-moderate extent of muscle damage [17]. Another recent systematic review pointed out the benefit of dietary protein in alleviating muscle damage [18], although it may not be beneficial to the recovery of physical performance [18,19]. Intriguingly, some of the protein hydrolysates exhibited beneficial effects on skeletal muscle, which were more effective than the constituent amino acid mixtures and/or intact proteins in animal models. This includes the stimulatory effect of whey protein hydrolysate on post-exercise muscle protein synthesis in rats [20], the preventive effects of whey protein hydrolysate on skeletal muscle loss induced by protein-free diet in rats [21], and the inductive effect of casein hydrolysate on mitochondrial biogenesis in mice [22]. Therefore, the involvement of protein-derived bioactive peptides in the hydrolysates is suggested. Thus, the intake of amino acids and protein (including protein hydrolysate) is closely tied to post-exercise adaptive responses of skeletal muscle tissue, which has a strong influence on physical performance.

Albumin is the major protein in serum and is exclusively synthesized in the liver, before being secreted into the circulation [23]. Similar to skeletal muscle protein, albumin synthesis is stimulated by dietary amino acids and protein [24,25,26], in addition to being responsive to exercise [27,28]. Due to its huge pool and long half-life [29], it has been proposed that albumin serves as a reservoir of excessive dietary amino acids that is protected from irreversible oxidation [24]. However, this notion might be negated by a study by Moore et al. [30]. In this study, both muscle protein and albumin synthesis exhibited a dose-dependent response to dietary protein ingestion, reaching a plateau at 20 g of ingestion and accompanied by stimulation of Leu oxidation (reflecting excessive protein intake). This suggests that the sequestration of excessive dietary amino acids in albumin as a reservoir may be minor compared with the large capacity of skeletal muscle. Nevertheless, serum albumin and its metabolism can be linked to skeletal muscle and its adaptive responses to exercise, as Visser et al. reported that lower serum albumin concentration in the elderly was associated with a greater loss of appendicular skeletal muscle mass during a five-year follow up, even after adjustment for confounders, such as plasma inflammation levels and protein intake [31].

It has recently been reported that protein and energy intake modulated the redox state of plasma albumin in rats [32,33]. The redox state of albumin correlated with albumin turnover, which was more responsive to protein intake compared with plasma albumin level [33]. Furthermore, the redox state of serum albumin was modulated by strenuous exercise [34,35,36,37]. Therefore, it can be posited that the redox state could be associated with the nutritional and physiological status before/after exercise in humans. This article provides an overview of serum albumin and its redox states, before discussing the potential role of the redox state of serum albumin in the context of amino acids/protein nutritional status and exercise.

## 2. Nutritional Regulation of Albumin Metabolism

Human serum albumin consists of a single polypeptide chain of 585 amino acid residues and has a molecular weight of approximately 66 kDa [23]. As discussed above, this protein is exclusively synthesized in the liver [23], and the synthesis is modulated by dietary factors, such as amino acid and protein intake [24,25,26,30]. Thus, serum albumin level has been widely used as a marker of protein nutritional status [38]. Nutritional regulation of albumin synthesis occurs primarily at the transcriptional level, but it is also modulated post-transcriptionally.

Hepatocyte nuclear factor 1 (HNF-1) is one of the transcriptional factors that strongly activate the transcription of albumin gene [39]. Binding of HNF-1 to the promoter of albumin gene was decreased in hepatoma cells when they were incubated with 5% albumin [40], while hepatic albumin gene expression was suppressed in rats intravenously infused with albumin [41]. One of the primary roles of serum albumin is to maintain serum colloidal osmotic pressure (COP) [23], and HNF-1-mediated feedback regulation is suggested to influence albumin synthesis in order to maintains COP homeostasis. In nutritional terms, this transcription factor is inactivated by directly binding to pyridoxal 5’-phosphate (PLP, the active form of vitamin B6) [42,43]. Furthermore, binding of HNF-1 to the promoter of albumin gene was attenuated and albumin gene expression was suppressed in the liver of rats maintained on an amino acid-depleted parenteral nutrition [44]. Hepatic PLP level was higher and pyridoxamine 5’-phosphate (PMP) level was lower in amino acid-depleted rats compared with amino acid-infused rats. Therefore, it was suggested that amino acid availability would modulate the hepatic PLP/PMP balance (they are converted to each other via transamination), with a higher PLP level downregulating albumin gene expression in amino acid-depleted rats. Thus, amino acid availability is likely responsible for HNF-1-mediated transcriptional regulation of the albumin gene.

Post-transcriptional regulation is considered another important part of albumin synthesis, which is also modulated by nutrients. The association of albumin mRNA with polysomes increased with graded levels of BCAAs that were administered intravenously in the liver of galactosamine-treated rats [45]. Binding of polypyrimidine tract-binding protein (PTB) to albumin mRNA was observed in the liver of the same rat model [46]. The level was higher when the animals were infused with standard amino acid formula than with BCAA-enriched formula, suggesting that the formation of albumin mRNA-PTB complex would hinder the association of polysomes to the mRNA and suppress its translation. It was also shown in human hepatoma cells that the intracellular localization of PTB was responsive to the composition of amino acids in the media [47]. When the cells were cultured in an amino acid-free medium, PTB was exported from nucleus to cytoplasm, where the complex of albumin mRNA-PTB was formed. This process was reversed when cells were cultured in an amino acid-complete medium. Following this, it was confirmed that albumin secretion was lower in the cells cultured in an amino acid-free medium than those in an amino acid-complete medium. Among the amino acids tested, leucine was responsible for the nuclear localization of PTB, which was inhibited by rapamycin treatment. Thus, leucine plays an important role in the regulation of PTB localization via mTOR signaling pathway, which consequently modulates hepatic albumin synthesis. However, the contribution of this machinery to albumin synthesis may be limited as dietary BCAA only slightly facilitated the translation of albumin mRNA in healthy rats [48,49].

It should be noted that albumin catabolism has not been extensively investigated in nutritional terms, when compared to albumin synthesis. However, it has long been known that the fractional catabolic rate of serum albumin decreased and its half-life extended under severe dietary protein restriction in humans and rats [50,51]. Therefore, it is likely that albumin catabolism would also be regulated by amino acid availability through novel molecular mechanisms.

## 3. Redox State of Serum Albumin

**Figure 1:**
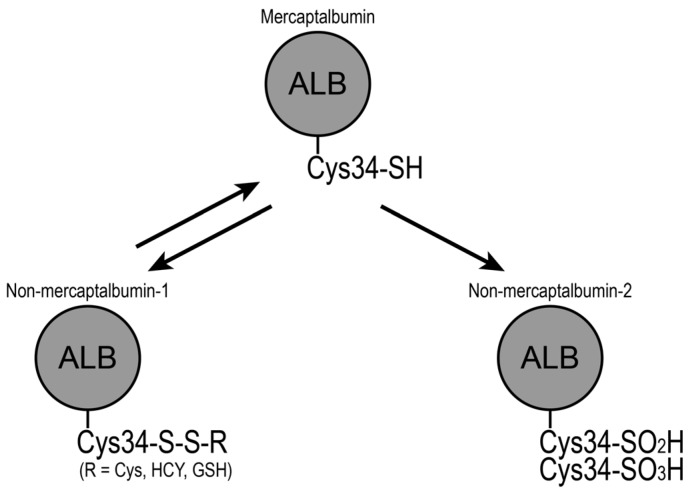
Scheme for the redox state of serum albumin. The free cysteine residue at position 34 (Cys34) of mercaptalbumin forms a disulfide bond with low molecular weight thiols such as cysteine (Cys), homocysteine (HCY), and glutathione (GSH). Alternatively, Cys34 of mercaptalbumin is oxidized to sulfinic acid or sulfonic acid. These oxidized forms of serum albumin are designated as non-mercaptalbumin-1 and -2, respectively.

Human serum albumin has 35 cysteine (Cys) residues. Although 34 Cys residues form intramolecular disulfide bonds, the remaining single Cys residue remains free at position 34 (Cys34) [23]. This free Cys34 is conserved in all mammals investigated and is involved in the heterogeneity of albumin isoforms. Albumin can be separated into three fractions on a Shodex-Asahipak ES-502N column, according to Cys34 status [52,53]: mercaptalbumin with reduced Cys34, non-mercaptalbumin-1 that has mixed disulfide on Cys34 with low molecular weight thiols such as Cys, homo-Cys or glutathione, and non-mercaptalbumin-2 with the thiol of Cys34 oxidized to sulfinic or sulfonic acid (Figure 1). Normally, mercaptalbumin constitutes the largest part of the three isoforms, accounting for > 70% in healthy adults [54]. However, the redox balance shifts to the oxidized state under several physiological and pathophysiological conditions.

The redox state of serum albumin has been extensively investigated in patients with liver failure. The shift to the oxidized state has been reported in patients with chronic liver diseases [55,56,57,58,59]. In particular, non-mercaptalbumin-2 markedly elevated with the progression of liver diseases [56,57]. This shift to the oxidized state is likely attributed to impaired albumin turnover and oxidative stress caused by hepatic disorders [60,61]. Oral supplementation with BCAA reversed the liver disease-related shift in rats and humans [59,62,63]. It can be speculated that supplementary BCAA not only serves as substrates for albumin synthesis, but also induces mTOR-mediated albumin translation as discussed above. This leads to the recovery of albumin turnover and the reversal of albumin redox state. Similarly, increased levels of oxidized serum albumin have also been observed in patients with renal diseases [64,65,66,67], where involvement of oxidative stress is suggested. The extent of oxidation correlated with renal function in non-dialysis patients [64], while hemodialysis reduced non-mercaptalbumin-1 levels in end stage renal disease patients [67]. Furthermore, the shift of the serum albumin redox state to the oxidized state has been found in diabetes [68,69], and ageing [70,71], which may also be explained by oxidative stress.

**Figure 2:**
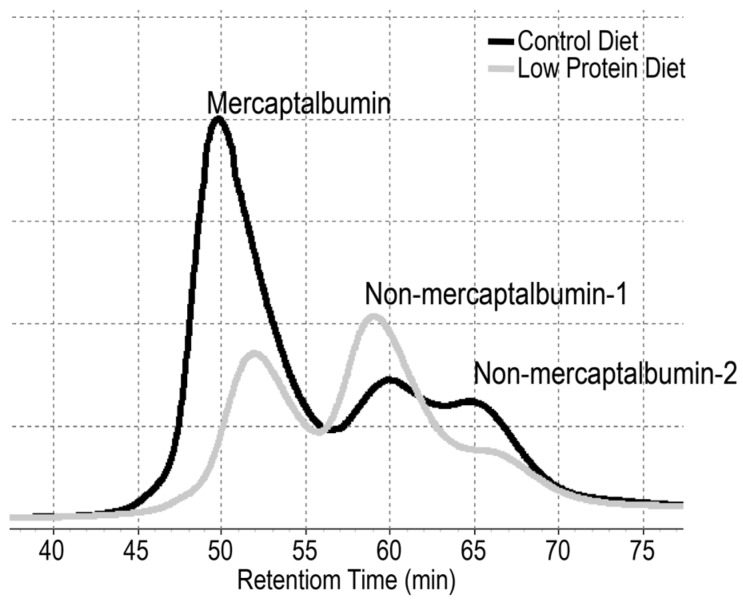
Chromatograms of albumin redox state. Albumin isoforms, mercaptalbumin, non-mercaptalbumin-1, and -2 can be separated chromatographically. Chromatograms of plasma albumin in rats fed control diet (black line) and a low protein diet (gray line) are shown (adapted from [33]). Compared with the control diet-fed rats, the low-protein diet-fed rats exhibited the shift of serum albumin redox state to the oxidized state.

As briefly described in the introduction section, recent animal studies have elucidated that albumin redox state is also influenced by dietary protein intake (Figure 2) [32,33]. The shift of plasma albumin redox state was found to correlate with albumin turnover and was therefore sensitive to protein nutritional status. Notably, this diet-induced shift did not parallel oxidative stress markers, such as thiobarbituric acid reactive substance and advanced oxidation protein product levels [33]. Clearly, the redox state of serum albumin is modulated not only by oxidative stress but also amino acid/protein nutritional status. Although clinical trials are warranted to substantiate the above notions, it may be applicable to humans as the shift of albumin redox state to the oxidized state was partially dissolved by BCAA supplementation in patients with liver disease, which supposedly occurred via improving albumin turnover [62,63]. It should be noted here that an increase in the ratio of non-mercaptalbumin-1 was reported in pregnant women with intrauterine growth restriction (IUGR) [72]. Although the authors attributed this increase to sustained oxidative stress associated with IUGR, this observation might indicate maternal protein/amino acid insufficiency, especially when considering the fact that lower amino acid availability is one of the greatest determinants to cause IUGR [73].

## 4. Redox State of Serum Albumin after Exercise and Its Potential Role

There has been limited investigation of the redox state of serum albumin in the context of post-exercise to date. Imai et al. first reported the shift of albumin redox state to the oxidized state in elite Kendo (Japanese fencing) athletes after intensive training camps [34,35]. A decrease in mercaptalbumin and increases in non-mercaptalbumin-1 and -2 were observed immediately after the end of the last training [34]. The albumin redox state returned back to normal on the following day after the training camp [35]. Lamprecht et al. subsequently reported the post-exercise shift of serum albumin redox state in Austrian policemen of a special anti-terrorism force, after they performed on a cycle ergometer at 70–80% VO_2max_ [36,37]. A decrease in mercaptalbumin and an increase in non-mercaptalbumin-1 were observed immediately and 30 min after the exercise. The shift proceeded in exercise-intensity dependent manner. The shift was reversed 30 hours after the exercise. Notably, contrary to the reports by Imai et al. [34], no significant change in non-mercaptalbumin-2 was observed [36]. As exercise is known to produce reactive nitrogen and oxygen species [74], these shifts of serum albumin redox state could be attributed primarily to exercise-induced oxidative stress. Moreover, as albumin is the most abundant serum protein and is constantly exchanged between blood circulation and interstitial fluid [23], the redox state of serum albumin could serve as a systemic redox marker after exercise training. Exercise-induced oxidative stress has a dichotomy. It has long been considered as detrimental to muscle fibers, manifesting as fatigue and muscle soreness. However, a body of recent investigation has elucidated that exercise-induced oxidative stress would be favorable and even required for post-exercise adaptation such as skeletal muscle hypertrophy and mitochondrial biogenesis [74]. Therefore, it can be speculated that assessing serum albumin redox state may help figure out the “optimal” extent of oxidative stress, or optimal bout of exercise, for skeletal muscle adaptation.

Effects of antioxidant supplementation on the serum albumin redox state were investigated by the same two research groups (although it has recently been acknowledged that antioxidant supplementation at times has a negative impact on post-exercise skeletal muscle adaptation [75]). Supplementation of propolis, an antioxidant derived from plant resins collected by honey bees, was effective in attenuating a decrease in mercaptalbumin during the Kendo training camp [35]. In contrast, long-term pre-exercise supplementation with a juice powder concentrate (fruit and vegetable extract) had no effect on reversing the shift of albumin redox state after the cycle ergometer performance [37]. Thus, studies on dietary antioxidant supplementation are limited and its effectiveness remains unclear. Contrary to antioxidant supplementation, no study has been reported to date regarding whether the redox state of serum albumin would be modulated by amino acids/protein nutritional status, including pre- and post-exercise supplementation. However, the report by Imai et al. is notable as the redox state of serum albumin was over-compensated a day after the Kendo training camp, with the percentage of mercaptalbumin being 72.6% before the camp, and 83.2% after the camp [35]. This over-compensation may suggest a post-exercise increase in albumin synthesis rate, as albumin synthesis is stimulated by exercise [27,28], and the mercaptalbumin ratio correlated with albumin turnover including albumin synthesis rate [33]. Furthermore, when considering that albumin synthesis rate is also stimulated by dietary amino acids/proteins [24,25,26], pre- and post-exercise measurement of the albumin redox state could help assess amino acid/protein requirement in athletes, which is likely to contribute to better post-exercise skeletal muscle adaptive responses. No study has been reported in this terms and further investigation is warranted.

## 5. Conclusions

**Figure 3:**
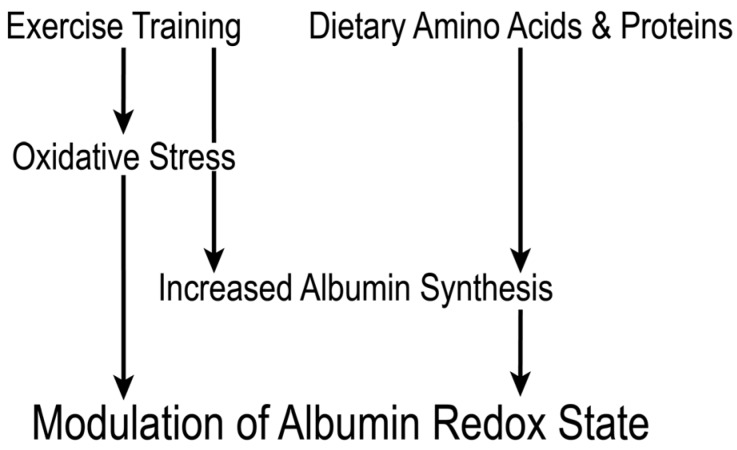
Proposed scheme for the redox state of serum albumin in the context of exercise training. Exercise modulates the redox state of serum albumin via inducing oxidative stress and increasing albumin synthesis. Amino acid/protein nutritional status also affects albumin redox state through albumin synthesis rate. Measurement of pre- and post-exercise albumin redox state would serve to assess amino acid/protein nutritional status and exercise-induced oxidative stress, which are closely associated with skeletal muscle adaptive responses.

This review extensively discussed serum albumin and its redox state in terms of nutrition and exercise. Particularly, the redox state of serum albumin is sensitively modulated by albumin turnover and oxidative stress. Therefore, the balance is responsive to amino acid/protein nutritional status and exercise training. It can be speculated that measurement of albumin redox state in athletes, before and after exercise, could serve to assess amino acid/protein nutritional status and exercise-induced oxidative stress, which are closely associated with skeletal muscle adaptive responses (Figure 3). However, studies on the association between albumin redox state and exercise are limited, and there is an array of multifaceted open questions, such as to what extent amino acid/protein nutritional status influences post-exercise albumin redox state, to what extent post-exercise shift of albumin redox state reflects exercise-induced oxidative stress, and whether post-exercise recovery of mercaptalbumin ratio correlates with skeletal muscle adaptive responses. Thus, extensive animal and clinical studies are warranted.
